# Changes in microbial community structure and yield responses with the use of nano-fertilizers of nitrogen and zinc in wheat–maize system

**DOI:** 10.1038/s41598-023-48951-3

**Published:** 2024-01-11

**Authors:** Pravin Kumar Upadhyay, Abir Dey, Vinod Kumar Singh, Brahma Swaroop Dwivedi, Rajiv Kumar Singh, G. A. Rajanna, Subhash Babu, Sanjay Singh Rathore, Kapila Shekhawat, Pradeep Kumar Rai, Nalini Kanta Choudhury, Neeraj Budhlakoti, Dwijesh Chandra Mishra, Anil Rai, Awtar Singh, Ajay Kumar Bhardwaj, Gaurav Shukla

**Affiliations:** 1 ICAR-Indian Agricultural Research Institute, New Delhi, 110 012 India; 2 ICAR-Central Research Institute for Dryland Agriculture, Hyderabad, 500 059 India; 3 Agricultural Scientist Recruitment Board, New Delhi, 110 012 India; 4 ICAR- Directorate of Groundnut Research, Regional Station, Ananthapur, 515 701 India; 5 IFFCO-Nano Fertilizer Plant, Phulpur Unit, Ghiyanagar, Prayagraj, Uttar Pradesh 212404 India; 6 ICAR-Indian Agricultural Statistics Research Institute, New Delhi, 110 012 India; 7 ICAR-Central Soil Salinity Research Institute, Karnal, 132001 India

**Keywords:** Plant ecology, Natural variation in plants

## Abstract

The growing popularity of nano-fertilization around the world for enhancing yield and nutrient use efficiency has been realized, however its influence on soil microbial structure is not fully understood. The purpose of carrying out this study was to assess the combined effect of nano and conventional fertilizers on the soil biological indicators and crop yield in a wheat–maize system. The results indicate that the at par grain yield of wheat and maize was obtained with application of 75% of recommended nitrogen (N) with full dose of phosphorus (P) and potassium (K) through conventional fertilizers along with nano-N (nano-urea) or nano-N plus nano-Zn sprays and N_100_PK i.e. business as usual (recommended dose of fertilizer). Important soil microbial property like microbial biomass carbon was found statistically similar with nano fertilizer-based management (N_75_PK + nano-N, and N_75_PK + nano-N + nano-Zn) and conventional management (N_100_PK), during both wheat and maize seasons. The experimental data indicated that the application of foliar spray of nano-fertilizers along with 75% N as basal is a sustainable nutrient management approach with respect to growth, yield and rhizosphere biological activity. Furthermore, two foliar sprays of nano-N or nano-N + nano-Zn curtailed N requirement by 25%, furthermore enhanced soil microbial diversity and the microbial community structure. The specific microbial groups, including *Actinobacteria*, *Bacteroidia*, and *Proteobacteria*, were present in abundance and were positively correlated with wheat and maize yield and soil microbial biomass carbon. Thus, one of the best nutrient management approaches for sustaining productivity and maintaining sound microbial diversity in wheat–maize rotation is the combined use of nano-fertilizers and conventional fertilizers.

## Introduction

Application of mineral fertilizers is the most common nutrient management practices^1^ for improving soil fertility^2^ and enhancing crop yield^3^. The use of intensive mineral fertilizers^4^ to the soil^5^ have been reported to cause environmental degradation^3,6^ through biodiversity loss^7,8^, nutrient runoff, leaching losses^9^, and water pollution. Under field conditions, nitrogen use efficiencies (NUE) of conventional fertilizers rarely exceed 30–35%^10^, while micronutrient use efficiency is even low, i.e. 2–5%^11^. Therefore, it is very important to protect and sustain long-term productivity of soils from improper management practices such as excessive and injudicious application of chemicals which lead to loss of soil microbial biodiversity and productivity of crops.

Recently, the Indian Farmers Fertiliser Cooperative (IFFCO) developed nano-fertilizers i.e. nano-N (nano-urea) and nano-Zn for foliar spray as a source of N and Zn nutrients, respectively. The developed nano-fertilizers was first tested under controlled conditions in laboratory and a few small-scale pot studies were conducted to check its effectiveness^12,13^. The efficacy of nano-N and nano-Zn was tested based on multi-location (11,000 locations) on several crops (94 crops) in different crop seasons, both by the researchers and progressive farmers in India. It was found that the application of nano-urea enhanced yields in wheat^14–16^ and maize^17^ across the tested locations. Nano-urea discharges nutrients in 40–50 days^18^, and it is applied on the leaves instead of soil; whereas conventional urea is applied in soil and discharges nutrients in 2–7 days^19^. Leaching and volatilization accounts for more than 70% of applied conventional urea and leaving only < 20%^20^ of applied amount available for plant uptake and growth. Nano-fertilizers release nitrogen 12 times slower than conventional fertilizers and thus is available for functional metabolic interaction for a longer time, and this can be one of the reasons for increased grain yields of crops^21^. It has also been reported that the uptake mechanism is also triggered by the application of nano-fertilizers as foliar spray^22,23^. The initial studies indicate a possibility of curtailing fertilizer doses with subsequent applications of nano-fertilizer after basal N application. Nevertheless, conjoint use of nano-fertilizers with conventional source of minerals fertilizers can also provide a balance between the immediate and long-term availability of N throughout the crop cycle, besides improving soil biodiversity.

Soil biochemical processes are greatly influenced by the microorganisms^24^. Soil microorganisms are responsible for the decomposition of soil organic matter and recycling of nutrients. Therefore, microbial diversity in the soil indirectly indicates the quality and overall health of the soil^25,26^. There are many studies which advocate that fertilizer management greatly influences the soil microbial diversity^1,27,28^.

However, the impact of nano-fertilizers on microbial properties is still elusive. Therefore, in this study, the microbial community structure based on high throughput sequencing technologies (Next Generation Sequencing) was used to study the effect of nano-fertilizers on soil microbial niche under wheat–maize ecosystem. The broader goal of the study was to elucidate the effect of nano-fertilizers on soil microbial biomass carbon (SMBC), microbial community diversity, and its inclusive impact on wheat and maize productivity. The current study involves multi-disciplinary efforts to understand the impact of nano-fertilizers on the composition of soil microbial niches. This study analyses the impact of nano-N and nano-Zn fertilization with variable conventional fertilizer N management on the microbial niches, abundance and diversity which plays pivotal role in nutrient cycling.

## Materials and methods

### Site description

Field experiments were conducted at the research farm (latitude 28^o^38′0838’’ north and longitude 77^o^09′1441’’ East) of ICAR-Indian Agricultural Research Institute, New Delhi to evaluate the performance of Nano-N (nano-urea) and Nano-Zn fertilizers on soil microbial community structure, yield and soil microbial biomass carbon (SMBC). The sandy loam soil of the experimental site was mildly alkaline (pH 8.22) and non-saline (EC 0.24 dS m^−1^). Topsoil (0–15 cm) contained 0.58% organic C, 272 kg ha^−1^ available N, 22.3 kg ha^−1^ available P, and 311 kg ha^−1^ available K. DTPA-extractable Zn contents in the soil was 0.84 mg kg^−1^.

### Experiment details and sample collection

**Figure 1:**
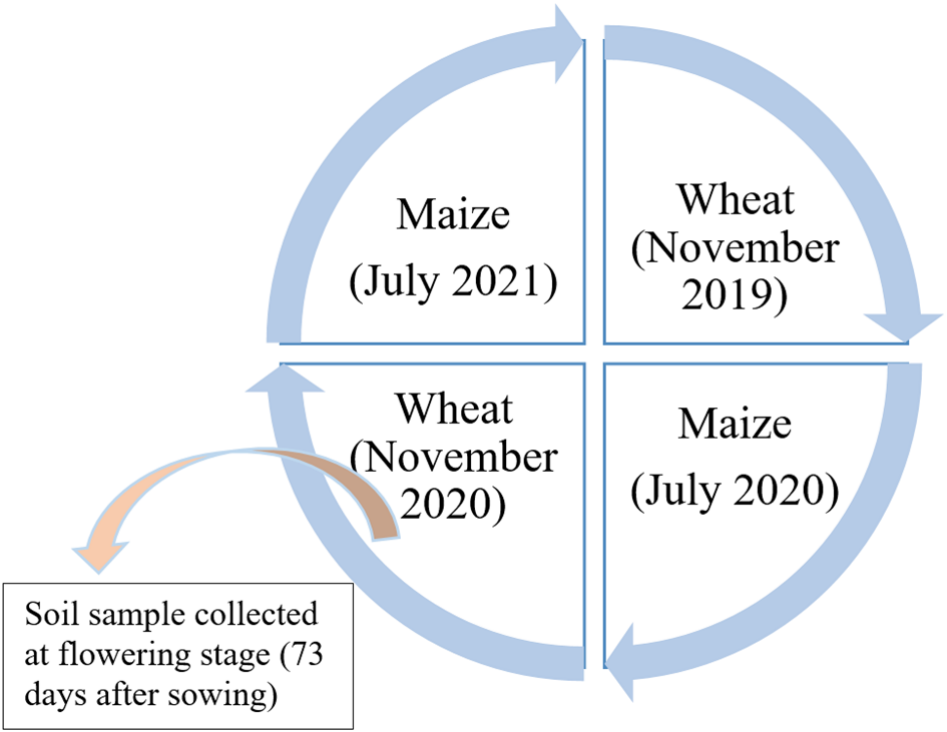
Crop cycle.

Experiments were conducted with 8 treatments (Table 1) in a randomized complete block design (RCBD) and replicated thrice. Fertilizer P and K were applied uniformly at recommended rates to all plots. Time of application of nano-N and nano-Zn in maize and wheat is given in Table 2. The details of package and practices followed during crop cycle are given in Table 3. Experiment was initiated with wheat crop in November 2019 followed by maize. The sampling was done at flowering stage of second cycle wheat crop (Fig. 1). A sterile shovel was pierced around the wheat plant up to a depth of 15 cm and dug out plant with its roots adhered with soil. Rhizosphere soil was collected from the rhizoplane region using sterile brushes. Similar procedures for rhizospheric soil collection were followed in different treatment plots. Soil samples collected from five different plants with 2 replicas of each treatments plot were thoroughly mixed and form a composite soil sample and stored at 4 °C in polypropylene sealed bags for further analysis. Soil microbial biomass carbon in soil samples was estimated as per the method of Ref.^29^.

**Table 1:** Treatments details of experiments undertaken in wheat-maize systems.

Symbol	Treatment	Treatment details
W1	N_0_PK	Recommended P and K (no-N)
W2	N_0_PK + Nano-N	Recommended P and K (no-N) + 2 nano-N sprays
W3	N_0_PK + Nano-N + Nano-Zn	Recommended P and K (no-N) + 2 nano-N sprays + 2 nano-Zn sprays
W4	N_100_PK	Recommended P, K and 100% of recommended N
W5	N_75_PK + Nano- N	Recommended P, K and 75% of recommended N + 2 nano-N sprays
W6	N_75_PK + Nano- Zn	Recommended P, K and 75% of recommended N + 2 nano-Zn sprays
W7	N_50_PK + Nano- N + Nano-Zn	Recommended P, K and 50% of recommended N + 2 nano-N sprays + 2 nano-Zn sprays
W8	N_75_PK + Nano-N + Nano-Zn	Recommended P, K and 75% of recommended N + 2 nano-N sprays + 2 nano-Zn sprays

*Note* Recommended fertilizer doses were 150 kg N ha^−1^, 75 kg P_2_O_5_ ha^−1^, 75 kg K_2_O ha^−1^ for maize and 120 kg N ha^−1^, 60 kg P_2_O_5_ ha^−1^, 60 kg K_2_O ha^−1^ for wheat crop.

**Table 2:** Time of application of nano-N and nano-Zn in different crops.

S.No	Crops	Date of sowing	Nano fertilizer	*1st spray*	*2nd spray*
1	Wheat (1st year)	08–11-2019	Nano-N and Zn	07–12-2019	07–01-2020
3	Wheat (2nd year)	05–11-2020	Nano-N and Zn	06–12-2020	11–01-2021
4	Maize (1st year)	11–07-2020	Nano-N and Zn	11–08-2020	04–09-2020
5	Maize (2nd year)	16–07-2021	Nano-N and Zn	16–08-2021	09–09-2021

**Table 3:** Agronomic package followed under different test crops.

Operation	Maize	Wheat
Tillage	Ploughing with cultivator (2 times), Double discing (1 time) and planking	Ploughing with cultivator (2 times), Double discing (1 time) and planking
Seed treatment	Thiram was used. Application rate was 2 g per kg seed	Thiram was used. Application rate was 2 g per kg seed
Variety/Hybrid	Pusa Jawahar Hybrid Maize 1	HD 3086
Seed rate	22 kg/ha	100 kg/ha
Weed management	Application of Pendimethaline as pre-emergence (1 l a.i./ha) + one hand weeding 22 days after sowing	Pre-emergence application of Pendimethaline @ 1 l a.i./ha + 75% Sulfosulfuron & 5% WG Metsulfuron@40 g a.i./ha
Insecticide	Emamectin benzoate 5 SG (0.4 ml/l) was used for management of fall army worm	–
Fungicide	–	–
Harvesting	Physiological maturity	Physiological maturity

### Nano fertilizers

Nano-fertilizers, nano-N (nano-urea) and nano-Zn were developed by the Indian Farmers Fertilizer Cooperative for use as an alternative to commercial fertilizers. Nano-urea contains functional nutrients derived primarily from urea which are treated with non-ionic surfactants and further stabilised in polymer matrices to produce nano clusters of less than 100 nm size. The fertilizer nano-urea has a size of particle in nanometre (nm) in one dimension (minimum 50% of the material), physical particle size ranging between 20 and 50 nm, and hydrodynamic particle size varying from 20 to 80 nm^18^. Nano-urea contains 4% N, has a shelf-life of about 2 years, and has a zeta potential > 30^18^. Nano-zinc (nano-Zn) is manufactured from the precursor salts of zinc which are further stabilised in polymer matrices to produce size less than 100 nm. It contains 10,000 ppm or 1% zinc.

### Soil DNA extraction, sequencing and preprocessing

The sample data was collected by using Power Soil DNA Isolation kit from each plot as per the manufacturer’s instructions. The quality and concentration of the soil DNA was measured by a Nano Drop 1000 spectrophotometer. The quality of the quantified DNA was then confirmed on the 1% agarose gel. The sequence libraries were prepared using Qubit 4.0 fluorometer using DNA HS assay kit, followed by PCR amplification of library. The samples were then sequenced on Ion 540 chip using 16S Ion Torrent Read Sequencing technique. Further, generated raw reads were quality checked using Fast QC v.0.11.9^30^ and summarized using Multi QC v.1.9^31^. The trimming (quality and adapter), filtering and masking of low quality of reads are performed using BB duk tool. The read data was reassessed post filtering using Fast QC and used in the downstream analysis.

### Processing and analysis of metagenomics sequence data

The merged metagenomics sequence reads were imported into QIIME2^32^ environment and de-replicated. These de-replicated sequences were clustered against SILVA database (available in QIIME2) at a similarity threshold of 99 percent, using closed-reference algorithm. Features that were present only in a single sample, annotated as mitochondria or chloroplast and remaining features were discarded. Further, maximum taxonomic abundance was estimated by the aid of the marker data profiling module of “MicrobiomeAnalyst”^33^. Here, the differential abundance analysis is performed using metagenome Seq v. 1.28.2. It is based on Moderated t test to study the difference of abundance.

### Microbial diversity analysis

**Figure 2:**
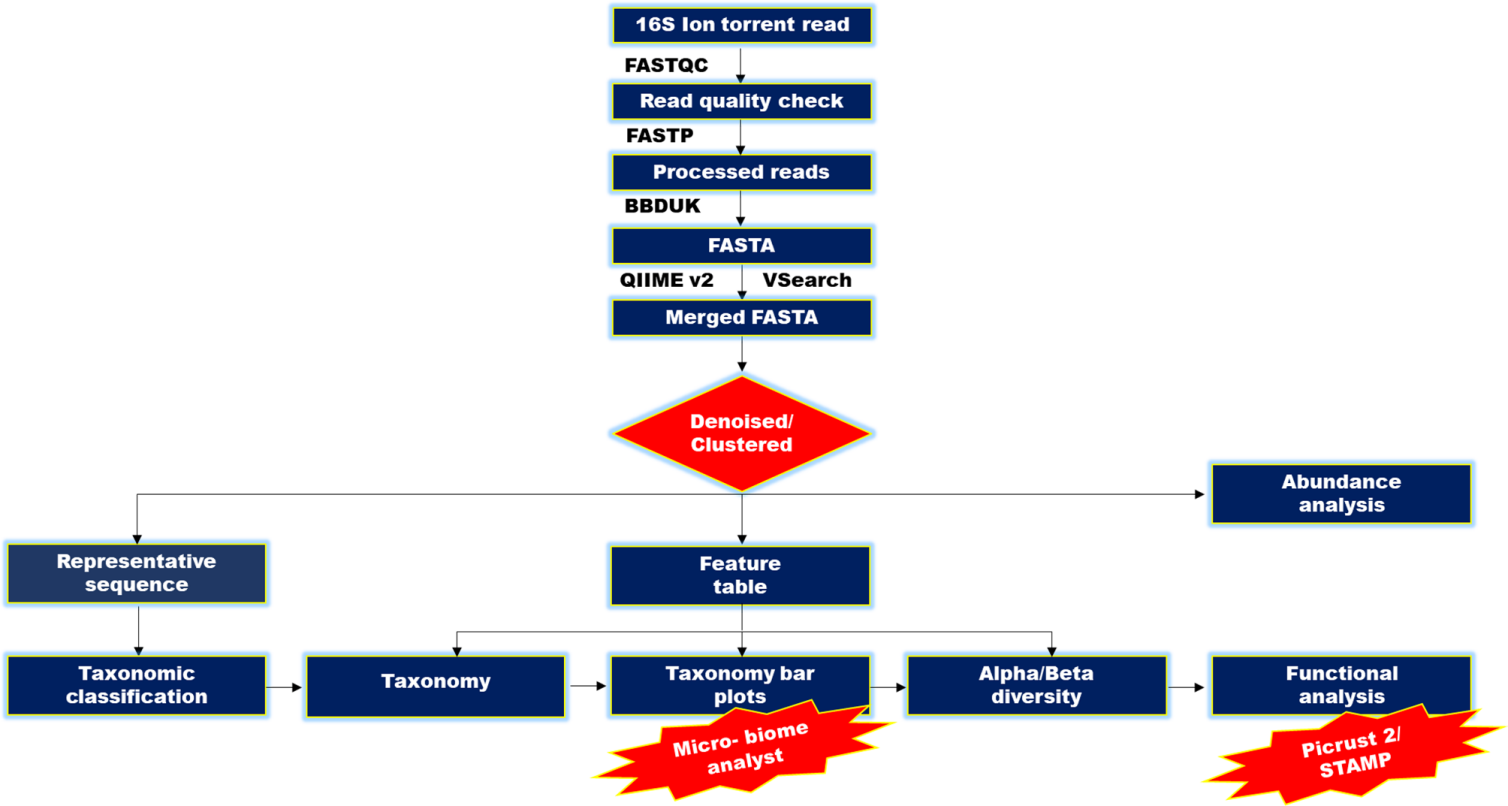
Overall analysis workflow.

The sequence number in the smallest library was used to narrow the filtered OTU (Operational Taxonomic Unit) table for analysis of Alpha and Beta diversity. Chao1 and abundance based coverage estimator (measure the species richness), and Shannon and Simpson (measure richness and distribution of taxa) indices were used for the estimation of alpha diversity using Ampvis2 R package (https://madsalbertsen.github.io/ampvis2/index.html). Principal co-ordinate analysis (PCoA) on bray–curtis distance matrics generated from the operational taxonomic units was used for the assessment of beta diversity. Microbial diversity is broadly categorized into six biological classification hierarchy of taxonomic groups i.e., phylum, class, order, family, genus and species to study their diversity in response to the different fertilizers and nano treatments. The microbial taxon is then used for detailed statistical analysis. The overall analysis workflow is given in Fig. 2.

### Differential microbial abundance analysis

The differential abundance analysis is used to identify highly significant microbes present in the samples. The 16S rRNA gene sequencing technique is the most common form of profiling of microbes and to study the relative abundance of different taxa present across different samples. The statistical assessment of functional profiles is carried out using STAMP (Statistical Analysis of Metagenomics Profile)^34^ software v 2.1.3. It is a Graphical User Interface (GUI) based software package implemented in python. It provides a range of statistical data analysis from simple exploratory plots to major statistical hypothesis testing. To study the conjoint effect of conventional fertilizers and nano-fertilizers (nano-N and nano-Zn) eight treatment combinations were taken (Table 1), and further we have divided them into four groups *v.i.z.* in first group, recommended phosphorous (P) & potassium (K) (No application of Nitrogen) (N_0_PK); and recommended P and K, and 100% of recommended N (N_100_PK). In the second group, recommended P and K (No application of N) + spray of nano-N (N_0_PK + Nano-N); and recommended P and K, and 75% of recommended N + spray of nano-N (N_75_PK + Nano- N). In the third group, recommended P and K (No application of N) + spraying of nano-N and nano-Zn (N_0_PK + Nano-N + Nano-Zn); recommended P and K, and 75% of recommended N + spraying of nano- Zn (N_75_PK + Nano- Zn). In the fourth group, recommended P and K, and 50% of recommended nitrogen + spraying of nano-N and nano- Zn (N_50_PK + Nano- N + Nano-Zn); and recommended P and K, and 75% of recommended N + spraying of nano-N and nano-Zn (N_75_PK + Nano-N + Nano-Zn) combination were taken and this group was created to analyze the effect of microbial diversity in 25% reduced nitrogen (N). To find significant microbes in the contrasting groups, two-sided Welch’s t-Test^35^ and Benjamini–Hochberg False Discover Rate (FDR) criteria were used, which is implemented in STAMP. Here, two group tests are taken to study the difference in mean proportion within the contrasting group. After running the Welch’s t-Test, specific features like significant microbes are filtered out by using p-value threshold of 0.05 (5% Level of Significance).

### Predictive functional analysis

Phylogenetic investigation of communities by reconstruction of unobserved stage 2 (PICRUSt2) was used for the analysis of predictive function of bacterial community^36^. The representative sequences were placed into a reference tree to predict the function of the bacterial communities. Castor was used for gene family prediction and further, multiple 16S rRNA gene copies were then normalized^37^. The predicted gene families were subsequently collapsed into MetaCyc pathway using MinPath^38^.

## Results

### Productivity of wheat and maize

**Figure 3:**
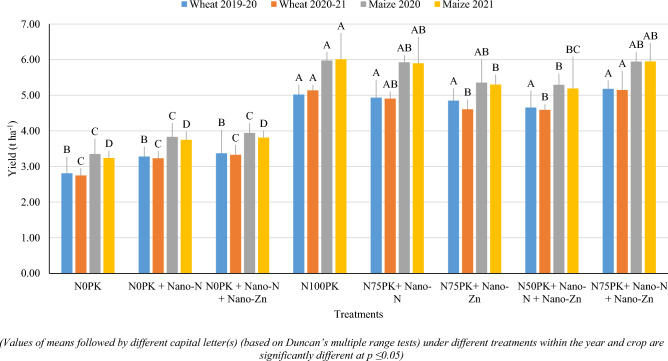
Effect of nano-N and nano-Zn on grain yield (t ha^-1^) of wheat and maize.

Conjoint application of conventional fertilizers (full dose of phosphorus and potassium, and graded level of nitrogen) along with nano fertilizers (nano-N and nano-Zn) significantly influenced the grain yield of wheat and maize during both the years (Fig. 3). Application of 75% of recommended N with full dose of PK along with nano-N and nano-Zn (N_75_PK + Nano-N + Nano-Zn) registered significantly higher yield of wheat (5.18 and 5.15 t ha^−1^ during first year and second year, respectively) over control [N_0_PK (2.81 and 2.75 t ha^−1^ during first year and second year, respectively), N_0_PK + Nano-N (3.28 and 3.23 t ha^−1^ during first year and second year, respectively), N_0_PK + Nano-N + Nano-Zn (3.37 and 3.33 t ha^−1^ during first year and second year, respectively)] (Fig. 3). However, N_75_PK + Nano-N + Nano-Zn was statistically at par with N_100_PK and N_75_PK + Nano- N with respect to grain yield of wheat during both the years. The reduction in the yield was 46.2, 37.0 and 35.1% in N_0_PK, N_0_PK + Nano-N and N_0_PK + Nano-N + Nano-Zn treatments over N_75_PK + Nano-N + Nano-Zn. It indicates application of nano-N and nano-Zn without N (through conventional fertilizers) could not suffice the requirement of the crops for getting optimum yield. Although, application of nano-N and nano-Zn (N_0_PK) have advantage over N_0_PK with respect to grain yield.

Application of 75% recommended N (112.5 kg N ha^−1^) + PK along with two sprays of Nano-N and nano-Zn recorded significantly higher grain yield over control [N_0_PK (3.35 and 3.24 t ha^−1^ during first year and second year, respectively), N_0_PK + Nano-N (3.83 and 3.75 t ha^−1^ during first year and second year, respectively), N_0_PK + Nano-N + Nano-Zn (3.94 and 3.81 t ha^−1^ during first year and second year, respectively)] and remained at par with N_100_PK and N_75_PK + nano-N (Fig. 3). Results revealed that application of nano-N, and nano-N and nano-Zn with conventional fertilizers have advantage over alone application of conventional fertilizers. Further, there is possibility of curtailing up to 25% of the recommended dose of N by application of nano-N and nano-Zn with conventional fertilizer.

### Soil microbial biomass carbon (SMBC)

**Figure 4:**
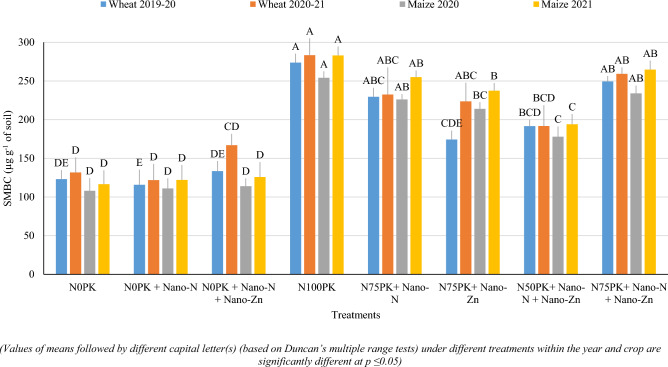
Effect of nano-N and nano-Zn on soil microbial biomass carbon (SMBC) under wheat and maize.

Soil microbial biomass carbon (SMBC) was monitored in soil at the flowering stage of both crops. Application of recommended N doses (N_100_PK) registered significantly higher SMBC in soil at flowering stage of wheat (274 and 283 µg g^−1^ of soil during 2019–20 and 2020–21, respectively) and maize (254 and 283 µg g^−1^ of soil during 2020 and 2021, respectively) compared with application of 50% of recommended N doses with Nano-N + Nano-Zn application (Fig. 4). On the other hand, treatments with application of 75% of recommended N doses with Nano- N alone or Nano-N + Nano-Zn registered similar values of SMBC as compared with N_100_PK treatments. Under nano-N or nano-n + nano-Zn spraying treatments with N_75_PK recorded significantly higher SMBC than that under N_0_PK, N_0_PK + Nano-N and N_0_PK + Nano-N + Nano-Zn during both the years in wheat and maize crops.

### Abundance analysis

**Figure 5:**
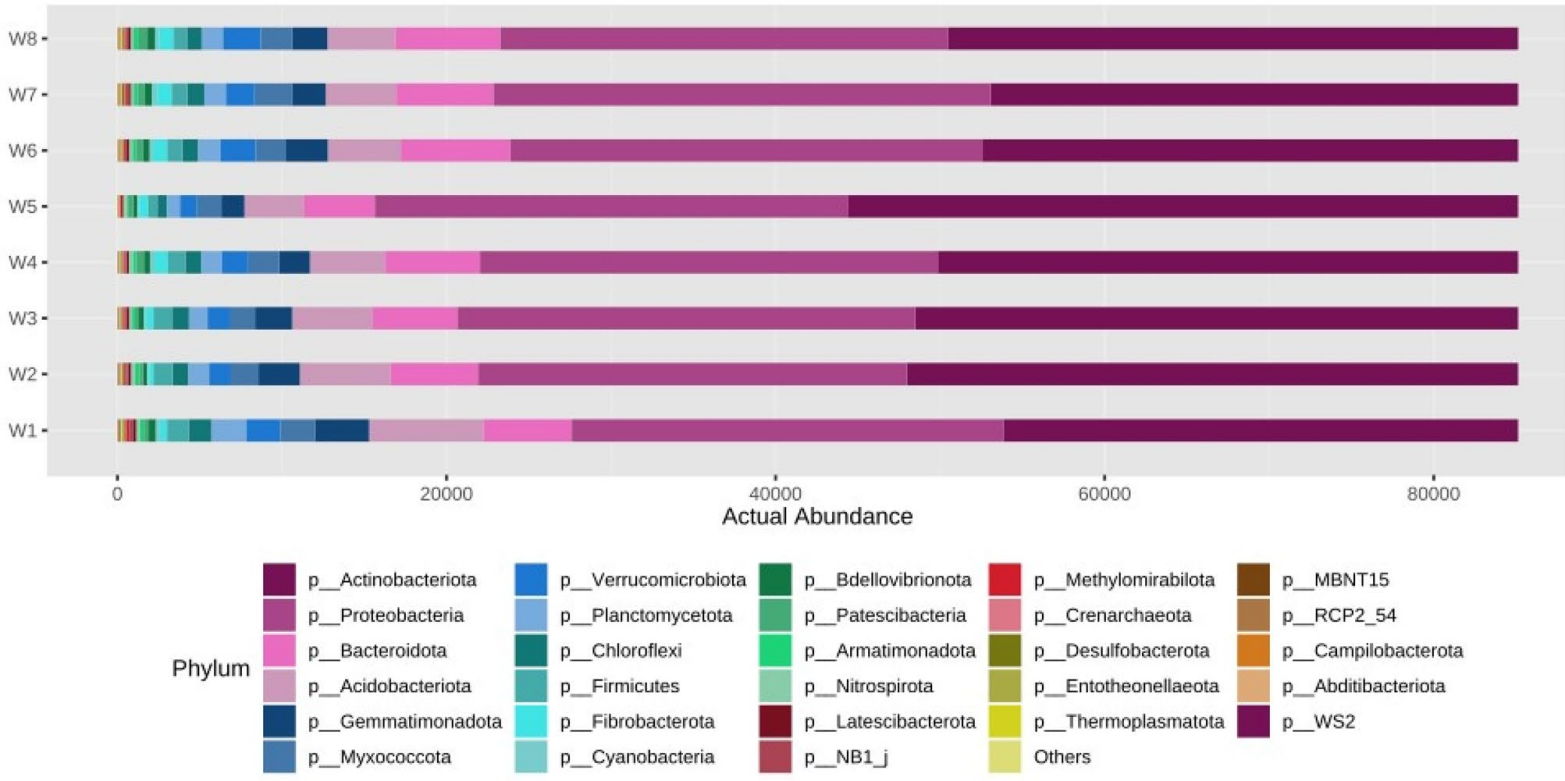
Actual abundance of different microbes at phylum level.

**Figure 6:**
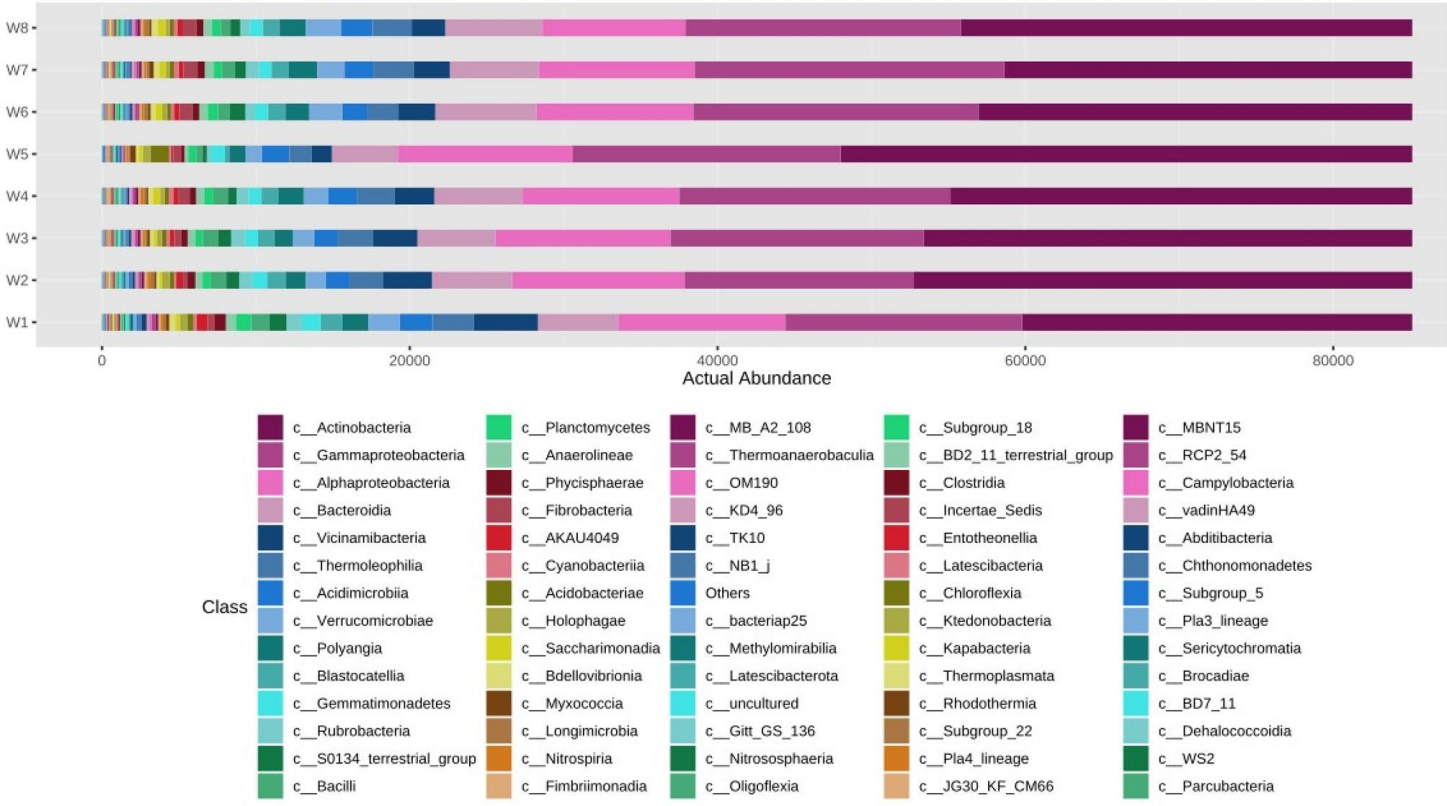
Actual abundance of different microbes at class level.

**Figure 7:**
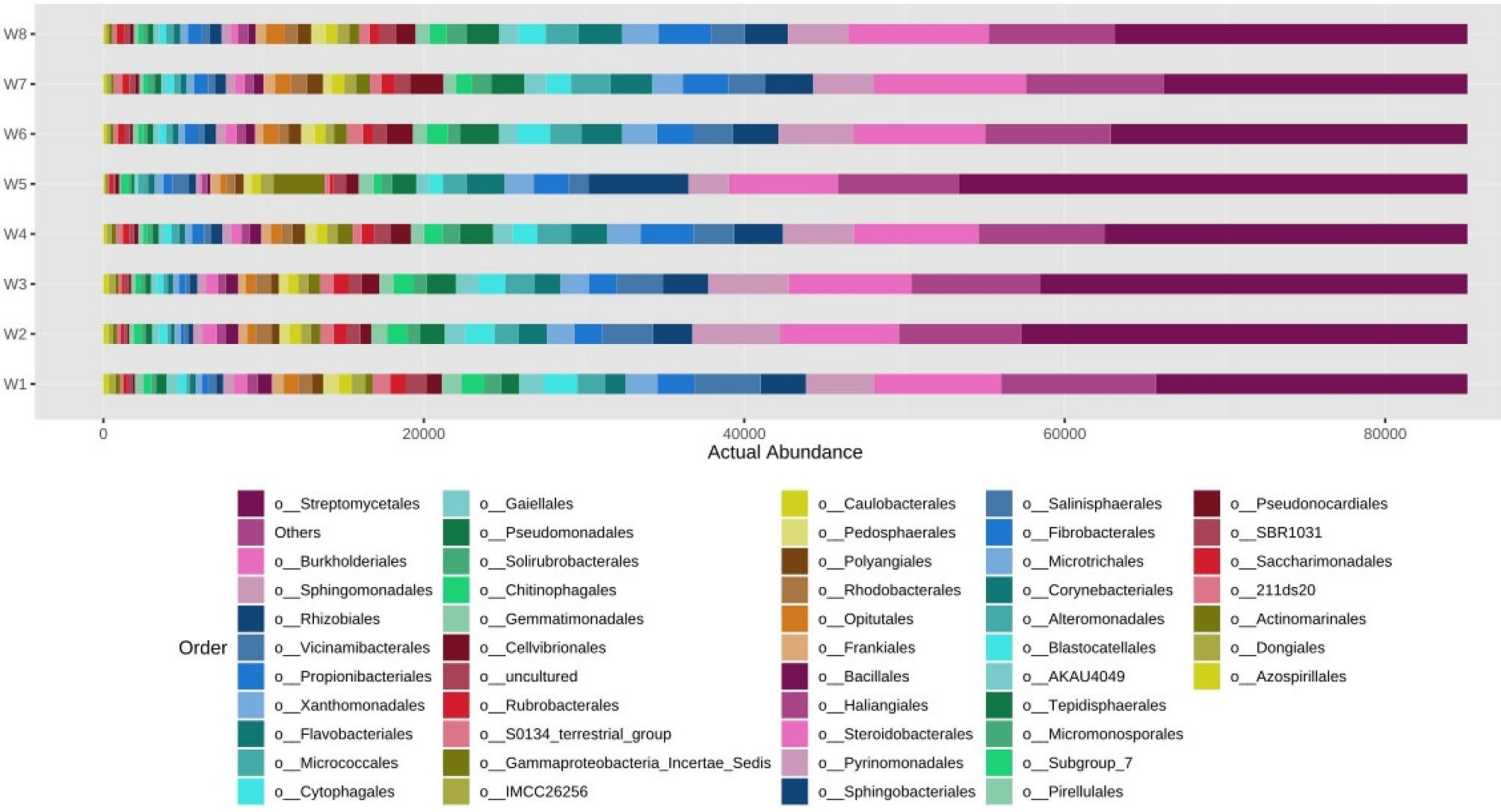
Actual abundance of different microbes at order level.

**Figure 8:**
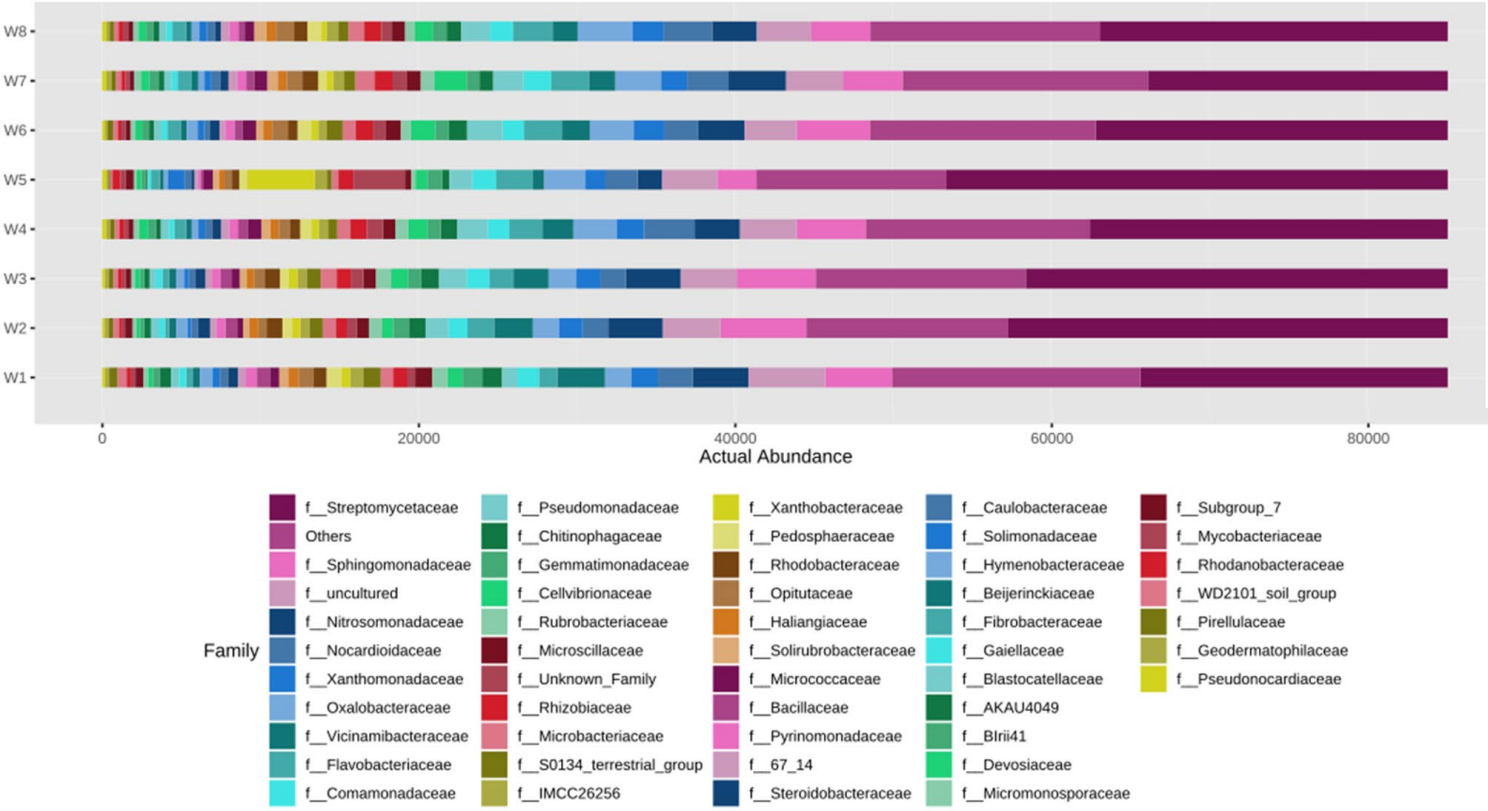
Actual abundance of different microbes at family level.

**Figure 9:**
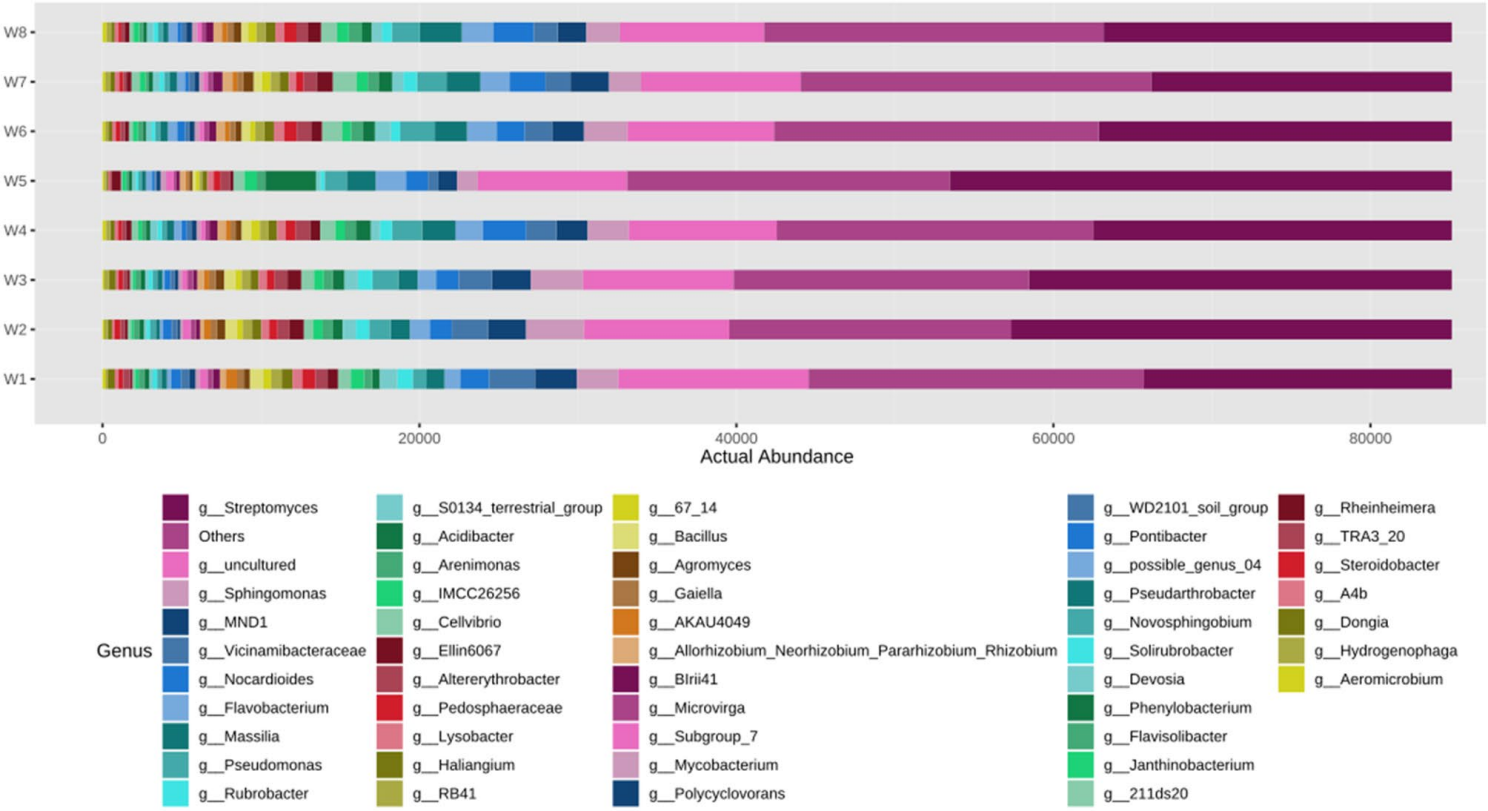
Actual abundance of different microbes at genus level.

**Figure 10:**
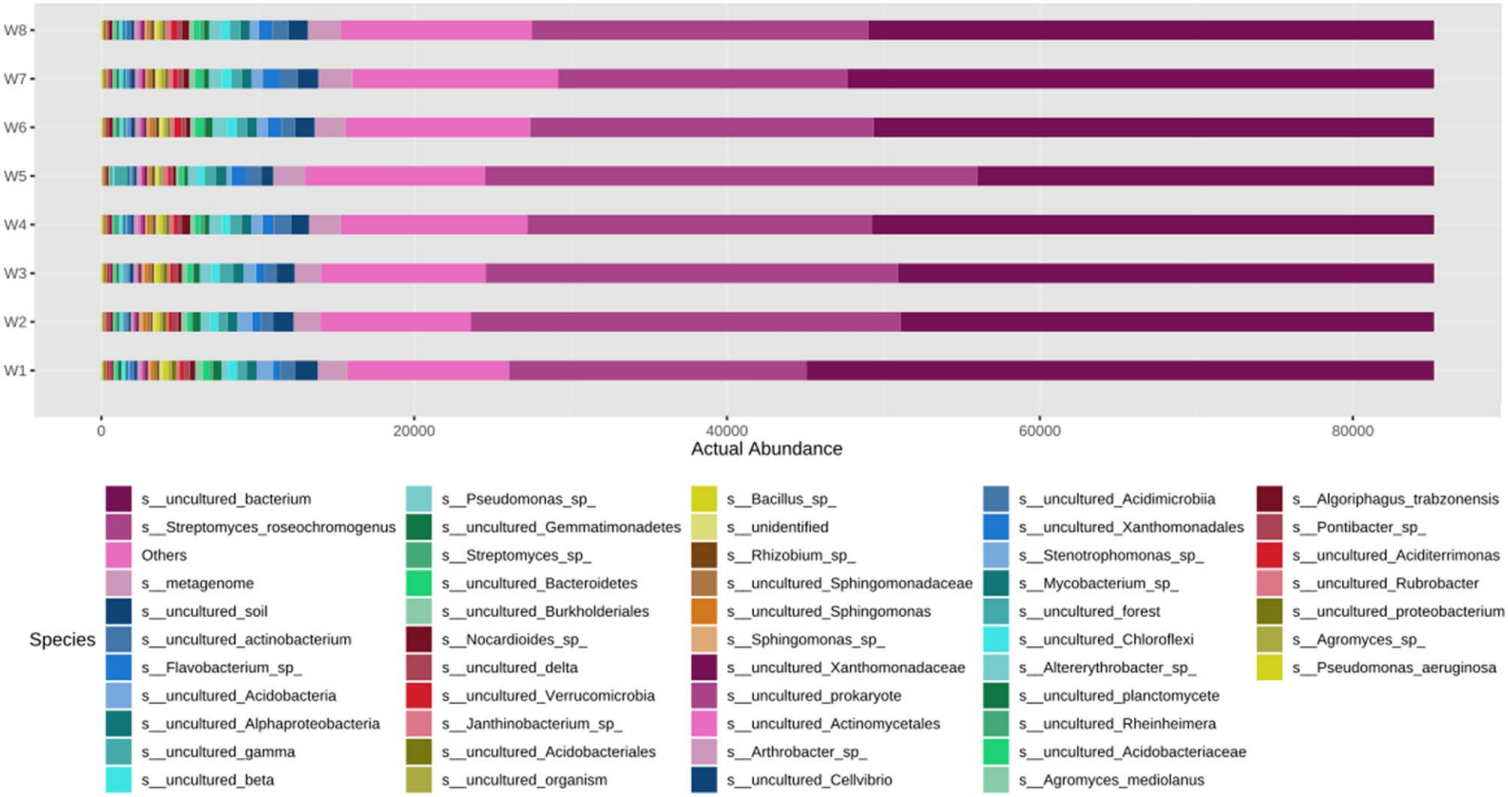
Actual abundance of different microbes at species level.

The abundance analysis was performed using metagenome Seq package and the results were given in Figs. 5, 6, 7, 8, 9, and 10 for phylum, class, order, family, genus, and species levels respectively.

The actual abundance of different microbes at phylum level was represented in Fig. 5. It was noticed that relative abundance of *Actinobacteriota* and *Proteobacteria* were significantly higher in comparison to other microbes present across eight soil samples. But its actual abundance varies from sample to sample. It was found that *Actinobacteriota* was more in case N_75_PK + Nano- N as compared to other treatments. The next most abundant microbe was *Proteobacteria* followed by *Bacteroidota, Acidobacteriota* etc. It was found that the actual abundance of *Actinobacteria* was the highest with respect to other organisms across all eight soil microbial samples at class level. But the most abundant *Actinobacteria* was found in N_75_PK + Nano- N (Fig. 6). The next three most abundant microbes at class levels were *Gammaproteobacteria* followed by *Alphaproteobacteria, Bacteroidia* etc.

Similarly at order level, it was noted that the actual abundance of *Streptomycetales* was found to be the highest among other microorganisms across all eight soil microbial samples (Fig. 7). But the abundance of *Streptomycetales* was found under N_75_PK + Nano- N as compared to other treatments. The Other most abundant microbes after *Streptomycetales* are *Burholderiales, Sphingomonadales* etc. respectively. At family level, *Streptomycetaceae* was the most abundant microbe found across all the soil microbial samples as shown in Fig. 8. Apart from *Strptomycetaceae; Sphingomonadaceae*, uncultured microbes, and *Nitromonadaceae* are other three abundant microbes found in abundant in chronological order. In Fig. 9, the most abundance microbe was *Streptomyces* followed by uncultured microbes, *Sphingomonas* and *MND1* microbes at genus level. At species level, uncultured bacterium followed by *Streptomyces roseochromogenus* and others were most abundantly organisms as shown in the Fig. 10.

### Microbial alpha diversity

**Figure 11:**
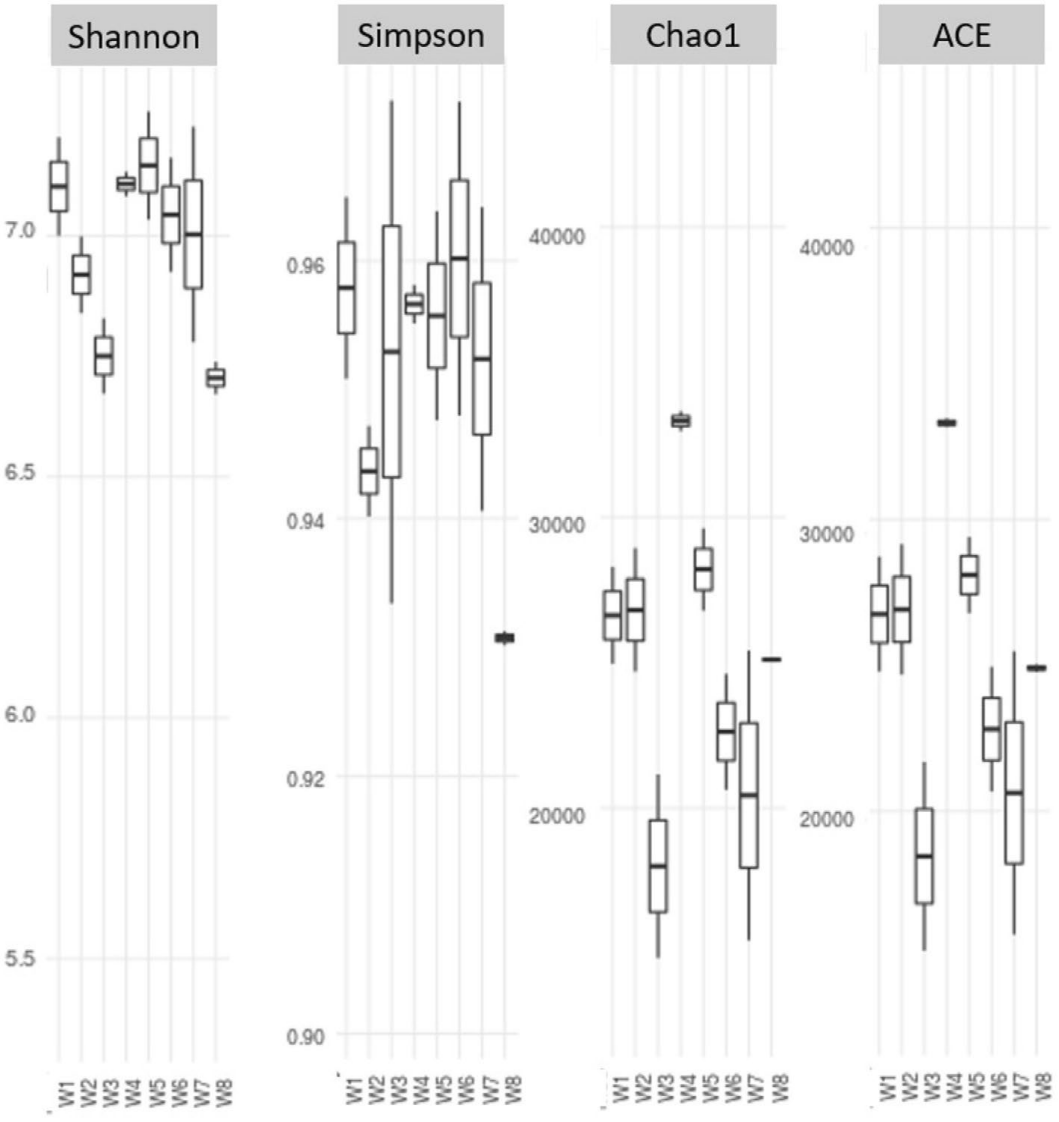
Alpha-diversity index measured using ACE, Chao1, Shannon, Simpson and Observed parameters in clockwise direction.

For each specimen, alpha diversity was estimated based on Shannon, Simpson, Chao1 and ACE indices, which measure the richness and distribution of taxa. All this was computed using the Ampvis-2 R package (https://madsalbertsen.github.io/ampvis2/index.html). Results of the same were presented in the Fig. 11. The microbial diversity as well as richness of several samples were quantified, and significant differences were observed among treatments. It was found that treatments N_100_PK (W4) and N75PK + Nano-N + Nano-Zn (W8) do not have much variation in comparison to other treatments based on all the indices. Whereas the treatments N_0_PK + Nano-N + Nano-Zn (W3) and N_50_PK + Nano- N + Nano-Zn (W7) are showing comparatively larger diversity and richness. These results indicate that application of Nano-Nitrogen and Nano-Zinc has significant impact on microbial diversity and their richness.

### Microbial beta diversity

**Figure 12:**
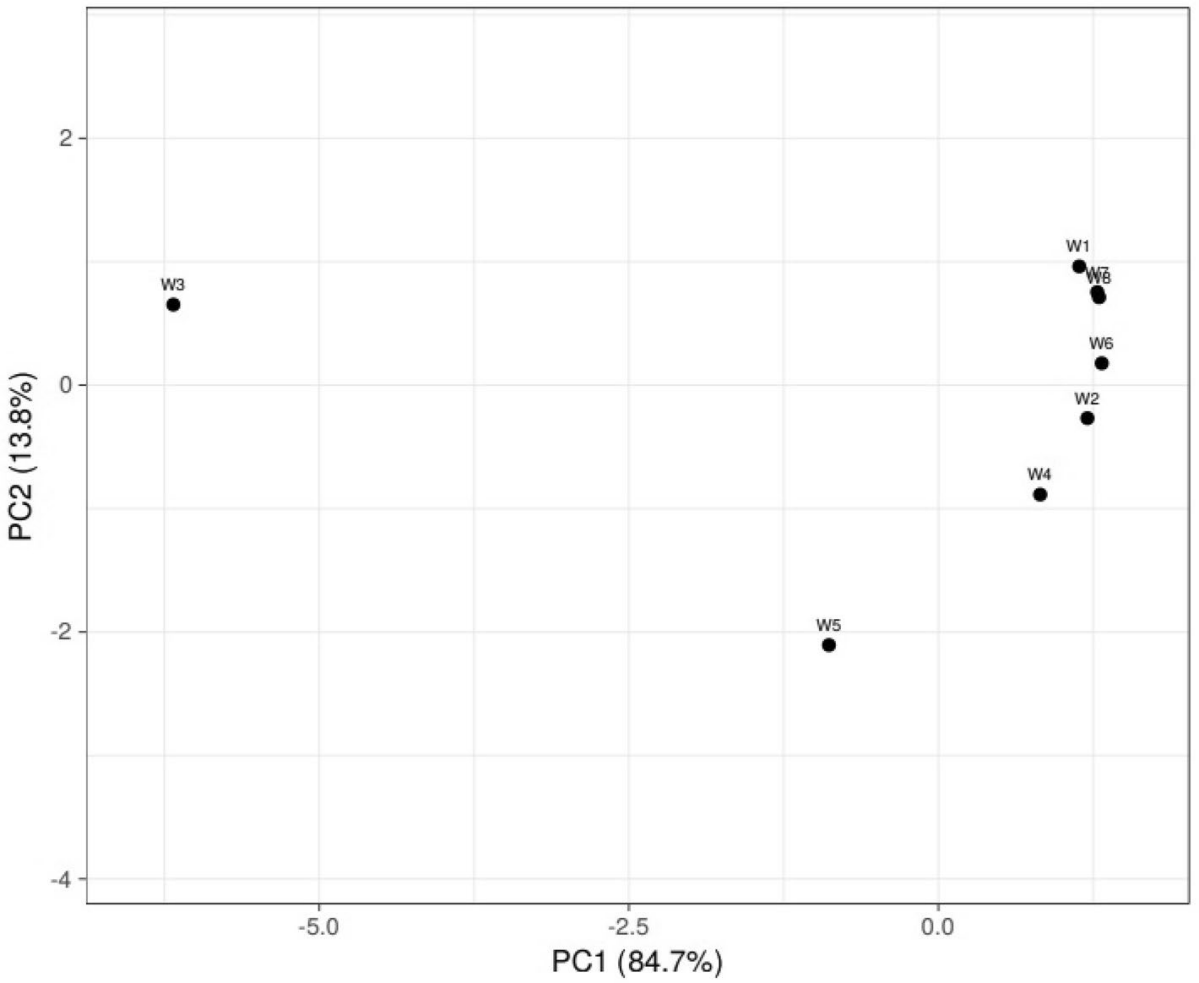
Beta Diversity Plot 3D at Genus Level.

Beta diversity (Fig. 12) was assessed using principal co-ordinate analysis (PCoA) on bray–curtis distance matrices generated using Operational Taxonomic Unit (OTU). The microbial beta diversity was calculated by using microbial communities present in the soil rhizosphere of wheat under different fertilizer treatments. Results indicate that PC1 explain most of the variations (84.7%) whereas PC2 explains (13.8%) present in the data. The analysis further confirmed that the microbial group of W1, W7, W8 and W2, W4, W6 treatments have similar pattern of microbial diversity in their respective groups. However, treatments W3 and W5 were clustered far apart from the other treatments indicating W3 and W5 have different microbial diversity pattern from remaining treatments (Fig. 12).

### Correlation among soil chemical properties and microbial community

The significant microbes which were found exclusively by the application of the nano-urea and nano-zinc are described in Table 4. It can be observed that, microbes found in the taxonomic groups were followed an upward triangle with highest number of microbes at the species level which was at the base of the triangle and lowest number of microbes classified at the phylum level. The bacteria of W2 were found to be significant at phylum level with the application of nano-urea. Similarly, at class level, polyangia, and cyanobactera were significant. The order level of taxonomic group has one significant microbes called *Micrococcales* which was found to be significant when comparing N_0_PK + Nano-N with N_75_PK + Nano- N. The family taxonomic group showed significant microbes at two combination of fertilizer application when N_0_PK + Nano-N vs N_75_PK + Nano- N and N_50_PK + Nano- N + Nano-Zn vs N_75_PK + Nano-N + Nano-Zn, the significant microbes present like *Micrococcaceae*; *Microscillaceae*, and *Xanthomonadaceae* respectively. Similar trend was seen at same two combination of fertilizer application when the comparisons made between N_0_PK + Nano-N vs N_75_PK + Nano- N and N_50_PK + Nano- N + Nano-Zn vs N_75_PK + Nano-N + Nano-Zn treatment combinations where significant microbes were *Pseudarthrobacter* and *Opitutus*, respectively. As the species level was the base level of the microbial taxonomic classification, therefore at all combination of the fertilizer application shows at least one significant microbe. *Janthinobacterium*_sp was found to be significant at N_0_PK + Nano-N vs N_75_PK + Nano- N combination; *Arthrobacter*_sp, and *Stenotrophomonas*_sp were found at N_0_PK + Nano-N vs N_75_PK + Nano- N fertilizer combinations; *Flavobacterium*_sp, and *Janthinobacterium*_sp were significantly associated to N_0_PK + Nano-N + Nano-Zn vs N_75_PK + Nano- Zn fertilizer combination. Unidentified species were most abundant in the N_50_PK + Nano- N + Nano-Zn vs N75PK + Nano-N + Nano-Zn treatment combinations. These microbes were then used to construct the heat map along with other microbes. The heat maps at three important levels like phylum, genus, and species were given in the Figs. 5, 9, 10, respectively. From Fig. 5, it was observed that actinobacteria and proteobacteria were amply present at phylum level but cyanobacteria whose abundance was less as compared to other two discussed above found to be significant when there was a 50% reduction of chemical nitrogen fertilizer, and that gap was filled by nano-urea. At genus level, *Streptomyces* bacteria was most abundantly found across all the wheat soil metagenome samples (Fig. 9). Other genus which were also abundantly found at genus level were *Sphingomonas*, MND1, *Nocardiodes*, and *Vicinamibacteraceae* etc. whereas, subgroup-2 followed by *Acidobacteria* were the most abundantly present under the treatment N_75_PK + Nano- N (W5). At Species level, *Streptomyces roseochromogenus* and some uncultured bacterium were most abundantly present in the soil microbial niche (Fig. 10).

**Table 4:** Effect of treatments on microbial diversity as per different taxonomic group.

Taxonomic group	Combination of fertilizer application	Number of significant microbes	Scientific name
Phylum	N_0_PK vs N_100_PK	0	–
	N_0_PK + Nano-N vs N_75_PK + Nano- N	1	WS2
	N_0_PK + Nano-N + Nano-Zn vs N_75_PK + Nano- Zn	0	–
	N_50_PK + Nano- N + Nano-Zn vs N_75_PK + Nano-N + Nano-Zn	1	Cyanobacteria
Class	N_0_PK vs N_100_PK	0	–
	N_0_PK + Nano-N vs N_75_PK + Nano- N	2	Polyangia, WS2
	N_0_PK + Nano-N + Nano-Zn vs N_75_PK + Nano- Zn	1	OM190
	N_50_PK + Nano- N + Nano-Zn vs N_75_PK + Nano-N + Nano-Zn	2	Cyanobacteria, JG30_KF_CM66
Order	N_0_PK vs N_100_PK	0	–
	N_0_PK + Nano-N vs N_75_PK + Nano- N	1	Micrococcales
	N_0_PK + Nano-N + Nano-Zn vs N_75_PK + Nano- Zn	0	–
	N_50_PK + Nano- N + Nano-Zn vs N_75_PK + Nano-N + Nano-Zn	0	–
Family	N_0_PK vs N_100_PK	0	–
	N_0_PK + Nano-N vs N_75_PK + Nano- N	1	Micrococcaceae
	N_0_PK + Nano-N + Nano-Zn vs N_75_PK + Nano- Zn	0	–
	N_50_PK + Nano- N + Nano-Zn vs N_75_PK + Nano-N + Nano-Zn	2	Microscillaceae, Xanthomonadaceae
Genus	N_0_PK vs N_100_PK	0	–
	N_0_PK + Nano-N vs N_75_PK + Nano- N	1	Pseudarthrobacter
	N_0_PK + Nano-N + Nano-Zn vs N_75_PK + Nano- Zn	0	–
	N_50_PK + Nano- N + Nano-Zn vs N_75_PK + Nano-N + Nano-Zn	1	Opitutus
Species	N_0_PK vs N_100_PK	1	Janthinobacterium_sp
	N_0_PK + Nano-N vs N_75_PK + Nano- N	2	Arthrobacter_sp, Stenotrophomonas_sp
	N_0_PK + Nano-N + Nano-Zn vs N_75_PK + Nano- Zn	2	Flavobacterium_sp, Janthinobacterium_sp
	N_50_PK + Nano- N + Nano-Zn vs N_75_PK + Nano-N + Nano-Zn	1	Unidentified_sp

## Discussion

### Productivity of wheat and maize

Application of 75% recommended dose of N + PK along with two sprays of nano-N or nano-N + nano-Zn recorded statistically at par results with N_100_PK for the yields of wheat and maize during both the years (Fig. 2). Hence, up to 25% of recommended N dose can be curtailed without any yield penalty, with nano-urea application. Whereas, the application of nano-N or nano-N + nano-Zn with full dose of PK had advantage over no application of nano-fertilizers (N_0_PK). In the current study, nano-N and nano-Zn were sprayed on leaves, leading to direct penetration through stomatal pores, and transportation through plasmodesmata^13^. Diminutive surface property and size of nano-urea enable its penetration into the plants via leaves. After entry in plant systems, nano-urea releases N in a controlled manner. Nano-urea boosts speedy nutrients availability to growing plant parts, ensuing increased dry matter accumulation, chlorophyll production, plant growth, development (data not reported) and yield. The yield of maize and wheat enhanced ideally owing to the synchronous release of nutrient from the nano-N and nano-Zn following crop’s demand^22^. Our results are consistent with^18^ who reported that foliar sprays of nano-fertilizer at critical crop growth stages either in isolation or in combination with fertilizers increases crop yields even at reduced levels of application of their conventional analogues. Al-Juthery et al. (2019)^15^ and Abdel-Aziz et al. (2016)^39^ indicated that foliar spray of nano-fertilizers significantly improved the plant growth characteristics and yield of wheat. Yield attributes viz., number of effective tillers per metre row length, ear length (cm), grains per ear, test weight etc. of crop were also higher in nano-fertilizer applied plots^40^. Nano-NPK applications were reported to stimulate the porphyrin molecules present in metabolic compounds, in turn, increasing plant biomass, yield and yield attributes of maize^23,23^. The yield enhancement due to Nano-fertilizers were reported in wheat^1,26,27^ and maize^17^ across the locations.

A complex relationship between soil microbial biomass carbon, wheat and maize yield, and microbial diversity, including the abundance of specific groups of microbes like *Actinobacteria*, *Bacteroidia*, *Streptomycetes*, and *Proteobacteria* was observed. Studies have shown that microbial biomass carbon, which represents the living fraction of organic matter in the soil, plays a crucial role in soil fertility and plant growth. This is because soil microorganisms are responsible for nutrient cycling and soil organic matter decomposition, making essential nutrients available to plants. Studies have revealed a positive correlation between wheat yield and microbial biomass carbon, as well as the abundance of specific microbial groups. For instance, *Actinobacteria*—known for producing antibiotics and stimulating plant growth, and *Bacteroidia*—involved in breaking down complex organic matter, have been found to be positively correlated with wheat and maize yield. Similarly, *Streptomycetes*, involved in the production of antibiotics and plant-growth promoting compounds, and *Proteobacteria*, involved in nitrogen fixation, also contribute to higher crop yields. In general, greater diversity of soil microbes, including the groups mentioned, is associated with improved soil fertility and higher crop yields^41^, highlighting the importance of microbial communities in sustainable agriculture^42^.

### Soil microbial biomass carbon

Application of recommended N doses promoted plant growth and biomass production. Greater root biomass under 100% N plots, paved the way for enhancement in biomass and activity of soil microbes in the vicinity of roots. The MBC varied in accordance with crop biomass yield. Improved biomass under N75PK + Nano-N, which was similar compared with 100% N plots, yielded similar values of MBC. On the other hand, there were no negative effect of nano-fertilizer spray on microbiological population, as often questioned upon.

### Soil microbial community structure

Application of Nano fertilizer on crops has both beneficial and deleterious effects on microorganisms which directly and indirectly affect the growth and development of plants^43^. The effect of nano fertilizer application on soil microbial diversity was studied by 16S soil metagenome through Ion torrent platform. Obtained sequences were analyzed through QIIME 2. Analysis revealed soil microbial diversity from genus to species level using Shanon index (species diversity), Simpson Index (Species diversity along with evenness of OTU), Chao-1 and ACE indices. Current study showed that crops exhibited more diverse soil microbes along the different combination of treatments. Treatment N_0_PK [W1], N_100_PK [W4], N_75_PK + Nano-N [W5], N_75_PK + Nano-Zn [W6], N_50_PK + Nano-N + Nano-Zn [W7] had no significant differences. On the contrary N0PK + Nano-N [W2], N0PK + Nano-N + Nano-Zn [W3] and N_75_PK + Nano-N + Nano-Zn [W8] had significantly different microbial population as compared to other counterparts. Chao-1 and ACE are non-parametric methods in order to identify rare species. In this observation what it could mean is “what are species that are rare compared to other treatments and uniquely enriched in a specific treatment”. A minor observation here was found that in case of samples rhizospheric soil, treatment N100PK [W4] showed more rare species identification by Chao-1 as compared to ACE index. In this study *Actinobacteriota* significantly differ along the different combination of treatment and similar study was reported by^44^ where they found that after the application of single walled carbon nanotube the relative abundance of *Proteobacteria* and *Bacteroidetes* increase, whereas abundance of *Actinobacteria* and *Chloroflexi* decrease. Similarly, most abundant microbes at class levels were *Gammaproteobacteria* followed by *Alphaproteobacteria, Bacteroidia* which is relevant with the findings of You et al. (2018). They concluded that after the application of Zinc oxide (ZnO) nanofertilizers (0.5–2 mg/g), the relative abundance of γ-*Proteobacteria*, α-*Proteobacteria* and *Bacilli* increase. Moreover, the actual abundance of *Streptomycetales* was also vary across the different combination of treatment at order level. In treatment combination of N75PK + Nano-N [W5], the abundance of *Actinobacteria and Streptomycetes* was found to be the highest as compared to other treatment combinations. Contrary to above findings Salas-Leiva et al. (2021) revealed that after the application of copper oxide (CuO) nano fertilizer (10–1000 mg/ kg) abundance of *Actinobacteria* and *Acidobacteria* were decreases. While after (50 mg/ kg) application of same nano fertilizer the population of *Sphingobacterium, Devosia, Pseudomonas, Rhizobium, Pseudoxanthomonas, Shinella, Dyadobacter* and *Pantoea were* increased. These microorganisms improved the nitrogen fixation and reduced denitrification process, resulting in an increase in the photosynthetic activity of plants. Similar contrast study was also reported in metallic silver^45,46^, Copper oxide (CuO)^47,48^, Titanium dioxide (TiO2)^49,50^ and Zinc oxide (ZnO)^51,52^ nano fertilizers. These study revealed that optimization of these nano fertilizers is very important prior to application in agriculture field.

Cyanobacteria, a gram negative bacteria which was significantly found at phylum level and at class level when there was a reduction of 50% nitrogen and that gap was filled by nano-urea. They actively participate in oxygenic photosynthesis, has a high biomass yield, growth on non-arable lands and a wide variety of water sources (contaminated and polluted waters), generation of useful by-products and bio-fuels, enhancing the soil fertility^53,54^.

A culture based approach was studied by Dhayalan et al.^55^ where they also found the similar result of increased microbial population in the treatment STCR (soil test crop response) based N as Urea (50%) and nano urea (2 sprays) as compared to rest of treatments. Further, effect of Zinc oxide nanofertilizers on microbial community structure was studied by You et al.^56^, and they revealed that after application of this nano fertilizers the relative abundance of *Proteobacteria* and *Bacilli* increased. In contrast to these studies, after the application of copper oxide (CuO)^57^ and titanium di oxide (TiO_2_)^49^ the relative abundance of *Actinobacteria* and *Acidobacteria* were decreased. Therefore, it is better to optimize these nanofertilizers prior to its application in agriculture fields.

## Conclusion

While fertilizers have greatly aided in increasing food production, their indecorous use has led to a decline in soil biodiversity. Combining nano-N/nano-Zn with traditional NPK fertilizers has been the subject of this research, and it has been found that this strategy can increase soil microbial biomass carbon and change the composition of the soil microbial communities by making it more diverse, all while reducing N usage by about 25% compared to the recommended dosage. These findings imply that nano-fertilizers may be a viable choice for sustainable agriculture due to their potential to cut down on nutrient loss, increase soil microbial diversity, and boost crop yield.

## Data Availability

The datasets generated and/or analysed during the current study are available in the [SRA data: PRJNA992358, submission ID: SUB13640894, release date: 2025-07-31] repository, [https://www.ncbi.nlm.nih.gov/sra/PRJNA992358]”.

## References

[1] Upadhyay PK (2019). Scientific validation of indigenous organic formulation-panchagavya for sustaining rice productivity and residual effect in rice-lentil system under hot semi-arid eco-region of middle Indo-Gangetic plains. Indian J. Tradit. Know..

[2] Upadhyay PK (2022). Soil health, energy budget, and rice productivity as influenced by cow products application with fertilizers under South Asian Eastern Indo-Gangetic Plains Zone. Front. Agron..

[3] Singh VK (2019). Yields, soil health and farm profits under a rice-wheat system: Long-term effect of fertilizers and organic manures applied alone and in combination. Agronomy.

[4] Dutta D (2022). Long-term impact of organic and inorganic fertilizers on soil organic carbon dynamics in a rice-wheat system. Land Degrad. Dev..

[5] Allam M (2022). Influence of organic and mineral fertilizers on soil organic carbon and crop productivity under different tillage systems: A meta-analysis. Agriculture.

[6] Ju XT, Kou CL, Christie P, Dou ZX, Zhang FS (2007). Changes in the soil environment from excessive application of fertilizers and manures to two contrasting intensive cropping systems on the North China Plain. Environ. Pollut..

[7] Qaim M, Sibhatu K, Siregar H, Grass I (2020). Environmental, economic, and social consequences of the oil palm boom. Annu. Rev. Resour. Econ..

[8] Jwaideh MAA, Sutanudjaja EH, Dalin C (2022). Global impacts of nitrogen and phosphorus fertiliser use for major crops on aquatic biodiversity. Int. J. LCA.

[9] Henryson K, Kätterer T, Tidåker P, Sundberg C (2020). Soil N_2_O emissions, N leaching and marine eutrophication in life cycle assessment-a comparison of modelling approaches. Sci. Total Environ..

[10] Al-Juthery HW, Lahmod NR, Al-Taee RA (2021). Intelligent, nano-fertilizers: A new technology for improvement nutrient use efficiency (article review). IOP Conf. Ser. Earth Environ. Sci..

[11] Baligar VC, Fageria NK, He ZL (2001). Nutrient use efficiency in plants. Commun. Soil Sci. Plant Anal..

[12] Raliya R, Saharan V, Dimkpa C, Biswas P (2017). Nanofertilizer for precision and sustainable agriculture: Current state and future perspectives. J. Agric. Food Chem..

[13] Kumar Y, Tiwari KN, Singh T, Raliya R (2021). Nanofertilizers and their role in sustainable agriculture. Ann. Plant Soil Res..

[14] Behboudi F, TahmasebiSarvestani Z, Kassaee MZ, ModaresSanavi SAM, Sorooshzadeh A (2018). Improving growth and yield of wheat under drought stress via application of SiO_2_ nanoparticles. J. Agri. Sci. Tech..

[15] Al-Juthery HW, Habeeb KH, Altaee FJK, Al-Taey DK, Al-Tawaha ARM (2019). Effect of foliar application of different sources of nano-fertilizers on growth and yield of wheat. Biosci. Res..

[16] Du W, Yang J, Peng Q, Liang X, Mao H (2019). Comparison study of zinc nanoparticles and zinc sulphate on wheat growth: From toxicity and zinc biofortification. Chemosphere.

[17] Manikandan A, Subramaniam KS (2018). Evaluation of zeolite-based nitrogen Nano-fertilizers 655 on maize growth, yield and quality on inceptisol and alfisols. Int. J. Plant Sci..

[18] Kumar Y, Singh T, Raliya R, Tiwari KN (2021). Nano fertilizers for sustainable crop production, higher nutrient use efficiency and enhanced profitability. Indian J. Fert..

[19] Seleiman MF (2021). Nano-fertilization as an emerging fertilization technique: Why can modern agriculture benefit from its use?. Plants.

[20] Kahrl F (2010). Greenhouse gas emissions from nitrogen fertilizer use in China. Environ. Sci. Policy.

[21] Saurabh K, Kanchikeri Math M, Datta SC, Thekkumpurath AS, Kumar R (2019). Nanoclay polymer composites loaded with urea and nitrification inhibitors for controlling nitrification in soil. Arch. Agron. Soil Sci..

[22] Babu S (2022). Nanofertilizers for agricultural and environmental sustainability. Chemosphere.

[23] Grillo R (2021). Ecotoxicological and regulatory aspects of environmental sustainability of nanopesticides. J. Hazard. Mater..

[24] Sekaran U, McCoy C, Kumar S, Subramanian S (2019). Soil microbial community structure and enzymatic activity responses to nitrogen management and landscape positions in switchgrass (*Panicumvirgatum* L.). Glob. Change Biol. Bioenergy.

[25] Zhong WH, Cai ZC (2007). Long-term effects of inorganic fertilizers on microbial biomass and community functional diversity in a paddy soil derived from quaternary red clay. Appl. Soil Ecol..

[26] Li J, Cooper JM, Li Y, Yang X, Zhao B (2015). Soil microbial community structure and function are significantly affected by long-term organic and mineral fertilization regimes in the North China Plain. Appl. Soil Ecol..

[27] Böhme L, Langer U, Böhme F (2005). Microbial biomass, enzyme activities and microbial community structure in two European long-term field experiments. Agric. Ecosyst. Environ..

[28] Zhang QC (2012). Chemical fertilizer and organic manure inputs in soil exhibit a vice versa pattern of microbial community structure. Appl. Soil Ecol..

[29] Wu J, Joergensen RG, Pommerening B, Chaussod R, Brookes PC (1990). Measurement of soil microbial biomass C by fumigation-extraction-an automated procedure. Soil Biol. Biochem..

[30] Andrews, S. FastQC: a quality control tool for high throughput sequence data. (2010).

[31] Ewels P, Magnusson M, Lundin S, Käller M (2016). MultiQC: Summarize analysis results for multiple tools and samples in a single report. Bioinformatics.

[32] Estaki M (2020). QIIME 2 enables comprehensive end-to-end analysis of diverse microbiome data and comparative studies with publicly available data. Curr. Protoc. Bioinform..

[33] Dhariwal A, Chong J, Habib S, King IL, Agellon LB, Xia J (2017). MicrobiomeAnalyst: A web-based tool for comprehensive statistical, visual and meta-analysis of microbiome data. Nucleic Acids Res..

[34] Parks DH, Tyson GW, Hugenholtz P, Beiko RG (2014). STAMP: Statistical analysis of taxonomic and functional profiles. Bioinformatics.

[35] Welch BL (1947). The generalization of ‘STUDENT'S’ problem when several different population varlances are involved. Biometrika.

[36] Douglas GM (2019). PICRUSt2: An improved and extensible approach for metagenome inference. Bio Rxiv.

[37] Louca S, Doebeli M (2018). Efficient comparative phylogenetics on large trees. Bioinformatics.

[38] Ye Y, Doak TG (2009). A parsimony approach to biological pathway reconstruction/inference for genomes and metagenomes. PLoS Comput. Biol..

[39] Abdel-Aziz HM, Hasaneen MN, Omer AM (2016). Nano chitosan-NPK fertilizer enhances the growth and productivity of wheat plants grown in sandy soil. Span. J. Agric. Res..

[40] Wu, M. Y. Effects of incorporation of nano-carbon into slow-released fertilizer on rice yield and nitrogen loss in surface water of paddy soil. In 2013 Third International Conference on Intelligent System Design and Engineering Applications. 676–681. 10.1109/ISDEA.2012.161 (2013**).**

[41] Upadhyay PK (2023). Conjoint application of nano-urea with conventional fertilizers: An energy efficient and environmentally robust approach for sustainable crop production. PlosOne.

[42] Zhang Y (2018). Long-term and legacy effects of manure application on soil microbial community composition. Biol. Fertil. Soils.

[43] Kalwani M, Chakdar H, Srivastava A, Pabbi S, Shukla P (2022). Effects of nanofertilizers on soil and plant-associated microbial communities: Emerging trends and perspectives. Chemosphere.

[44] Wu F (2019). Effects of various carbon nanotubes on soil bacterial community composition and structure. Environ. Sci. Technol..

[45] Nawaz S, Bano A (2020). Effects of PGPR (*Pseudomonas*
*sp.*) and Ag-nanoparticles on enzymatic activity and physiology of cucumber. Recent Pat. Food Nutr. Agric..

[46] Zhang W, Yu C, Wang X, Hai L (2020). Increased abundance of nitrogen transforming bacteria by higher C/N ratio reduces the total losses of N and C in chicken manure and corn stover mix composting. Bioresour. Technol..

[47] Guan X (2020). CuO nanoparticles alter the rhizospheric bacterial community and local nitrogen cycling for wheat grown in a calcareous soil. Environ. Sci. Technol..

[48] Salas-Leiva J (2021). Copper oxide nanoparticles slightly affect diversity and metabolic profiles of the prokaryotic community in pecan tree (*Caryaillinoinensis*) rhizospheric soil. Appl. Soil Ecol..

[49] Moll J (2017). Effects of titanium dioxide nanoparticles on soil microbial communities and wheat biomass. Soil Biol. Biochem..

[50] Palmqvist NGM, Bejai S, Meijer J, Seisenbaeva GA, Kessler VG (2015). Nano titania aided clustering and adhesion of beneficial bacteria to plant roots to enhance crop growth and stress management. Sci. Rep..

[51] Sharifi R, Khorramdel S (2016). Effects of nano-zinc oxide and seed inoculation by plant growth promoting rhizobacteria (PGPR) on yield, yield components and grain filling period of soybean (*Glycine*
*max* L.). Iran. J. Field Crops Res..

[52] Oghenerume P, Eduok S, Ita B, John O, Basssy I (2020). Impact of zinc oxide nanoparticles amended organic manure on Arachishypogaea growth response and rhizosphere bacterial community. Int. J. Plant Sci..

[53] Singh JS, Kumar A, Rai AN, Singh DP (2016). Cyanobacteria: A precious bio-resource in agriculture, ecosystem, and environmental sustainability. Front. Microbiol..

[54] Rai PK, Rai A, Sharma NK, Singh S (2018). Study of soil cyanobacteria along a rural-urban gradient. Algal Res..

[55] Dhayalan SA, Davamani V, Maheswari M, Maragatham S, Rahale CS (2023). Influence of nano urea on growth and microbial population in paddy ecosystem. Int. J. Environ. Clim..

[56] You T, Liu D, Chen J (2018). Effects of metal oxide nanoparticles on soil enzyme activities and bacterial communities in two different soil types. J. Soils Sediments.

[57] Salas-Leiva J, Salas-Leiva DE, Tovar-Ramírez D, Herrera-Pérez G, Tarango-Rivero S, Luna-Velasco A, Orrantia-Borunda E (2021). Copper oxide nanoparticles slightly affect diversity and metabolic profiles of the prokaryotic community in pecan tree (*Carya illinoinensis*) rhizospheric soil. Appl. Soil Ecol..

